# Alcohol related hepatitis in intensive care units: clinical and biological spectrum and mortality risk factors: a multicenter retrospective study

**DOI:** 10.1186/s13613-025-01450-2

**Published:** 2025-04-15

**Authors:** Maxime Gasperment, Léa Duhaut, Nicolas Terzi, Côme Gerard, Luc Haudebourg, Alexandre Demoule, Mialy Randrianarisoa, Vincent Castelain, Sacha Sarfati, Fabienne Tamion, Charlene Le Moal, Christophe Guitton, Gabriel Preda, Arnaud Galbois, Thibault Vieille, Gaël Piton, Marika Rudler, Guillaume Dumas, Hafid Ait-Oufella

**Affiliations:** 1 Service de Médecine Intensive-Réanimation, Hôpital Saint-Antoine, Assistance Publique-Hôpitaux de Paris, 184 Rue du Faubourg Saint-Antoine, Paris Cedex 12, 75571 France; 2 Service d’Hépatologie, Hôpital Paul Brousse, Assistance Publique-Hôpitaux de Paris, Villejuif, France; 3 Service de Médecine Intensive-Réanimation, CHU Grenoble Alpes, Université Grenoble-Alpes, Grenoble, France; 4 Service de Médecine Intensive-Réanimation, Groupe Hospitalier Pitié-Salpêtrière, Assistance Publique-Hôpitaux de Paris, Paris, France; 5 Service de Médecine Intensive-Réanimation, Hôpital de Hautepierre, Hôpitaux Universitaires de Strasbourg, Strasbourg, France; 6 Service de Médecine Intensive-Réanimation, Hôpital Charles Nicolle, Rouen, France; 7 Service de Réanimation médico-chirurgicale, Hôpital du Mans, Le Mans, France; 8 Service de Médecine Intensive-Réanimation, Hôpital Delafontaine, Saint-Denis, France; 9 Service de Réanimation polyvalente, Hôpital privé Claude Galien, Quincy Sous-Sénart, France; 10 Service de Médecine Intensive-Réanimation, Hôpital Universitaire de Besançon, Besançon, France; 11 Service d’Hépato-Gastroentérologie, Groupe Hospitalier Pitié-Salpêtrière, Assistance Publique-Hôpitaux de Paris, Paris, France; 12 Sorbonne Université, Paris, France

**Keywords:** Alcohol related hepatitis, Alcoholic hepatitis, Corticosteroids, Intensive care, Lille score, Liver transplantation, Mortality

## Abstract

**Background:**

Alcohol related hepatitis is responsible for high morbidity and mortality, but little is known about the management of patients with hepatitis specifically in intensive care units (ICU).

**Methods:**

Retrospective study including patients with alcohol related hepatitis hospitalized in 9 French ICUs (2006–2017). Alcohol related hepatitis was defined histologically or by an association of clinical and biological criteria according to current guidelines.

**Results:**

187 patients (median age: 53 [43–60]; male: 69%) were included. A liver biopsy was performed in 51% of cases. Patients were admitted for impaired consciousness (71%), sepsis (64%), shock (44%), respiratory failure (37%). At admission, median SOFA and MELD scores were 10 [7–13] and 31 [26–40] respectively. 63% of patients received invasive mechanical ventilation, 62% vasopressors, and 36% renal replacement therapy. 66% of patients received corticosteroids, and liver transplantation was performed in 16 patients (8.5%). ICU and in-hospital mortality were 37% and 53% respectively. By multivariate analysis, ICU mortality was associated with SOFA score (without total bilirubin) (SHR 1.08 [1.02–1.14] per one-point increase), arterial lactate (SHR 1.08 [1.03–1.13] per 1 mmol/L) and MELD score (SHR 1.09 [1.04–1.14] per 1 point), while employment was associated with increased survival (HR 0.49 [0.28–0.86]). After propensity score weighting, the use of corticosteroids did not affect ICU mortality in the overall population but had a beneficial effect in the subgroup of patients with histological proof. Patient prognosis was also better in responders assessed by Lille score at day 7 (OR 6.67 [2.44–20.15], *p* < 0.001).

**Conclusion:**

Alcohol related hepatitis is a severe condition leading to high mortality in ICU patients. Severity of organ failure at admission are mortality risk factors. Outcome was significantly better in responders to corticosteroids therapy according to Lille score. Early referral to tertiary centers to discuss liver transplantation should more widely be considered.

**Supplementary Information:**

The online version contains supplementary material available at 10.1186/s13613-025-01450-2.

## Background

Cirrhosis is the end stage of chronic liver disease, responsible for 170 000 deaths each year in Europe [1]. Its leading cause is chronic alcohol consumption. Alcohol related hepatitis (ARH) is a clinical syndrome characterized by a rapid onset of jaundice associated with liver and systemic inflammation that may lead to liver failure, with a mortality rate of up to 25% at one month in severe cases [2]. Alcohol related hepatitis occurs after a history of heavy drinking in patients usually at the cirrhosis stage. Biological markers include elevated serum bilirubin with moderately increased transaminase levels with an aspartate aminotransferase (AST) to alanine aminotransferase (ALT) ratio > 1.5. Elevated INR (International Normalized Ratio) and neutrophilia are also frequently found [3, 4]. Liver biopsy can be performed to confirm the diagnosis. While not mandatory, it is recommended in US guidelines in case of potential confounding factors [3]. Scoring systems used to assess patient severity and prognosis include Maddrey’s discriminant function and the Model for End-stage Liver Disease (MELD) score.

Specific treatment options for patients with alcohol related hepatitis are quite scarce: on top of alcohol abstinence, additional treatment options only discussed in severe cases (Maddrey score ≥ 32) include corticosteroids and liver transplantation. Treatment with corticosteroids increases short-term survival [5] but lacks long-term survival benefit [6]. The Lille model was therefore developed to early (day 7) distinguish patients responding to corticosteroids who will benefit from a full 28-day treatment from those who won’t [7, 8]. In case of lack of response to corticosteroids, liver transplantation shows interesting results in selected patients [9, 10].

Few studies specifically characterize patients admitted to ICUs with severe alcohol related hepatitis. Our study therefore aimed to describe the clinical and biological characteristics of critically ill patients admitted to ICUs for severe alcohol related hepatitis, to describe patient management, and to analyze mortality risk factors.

## Methods

### Study design

Between January 2006 and December 2017, all adult patients (≥ 18 years of age) admitted to nine French ICUs for alcohol related hepatitis were identified by searching hospital databases for codes K701 (alcoholic hepatitis) or K704 (alcoholic hepatic failure) as a principal or associated diagnosis, according to the International Classification of Diseases (10th version). Each medical record was then reviewed to confirm alcohol related hepatitis diagnosis, which was defined by histological proof on liver biopsy when performed (showing macrovesicular steatosis, with ≥ 1 of the following: neutrophil infiltration, hepatocyte injury (ballooning), and Mallory-Denk bodies), and in other cases the association of clinical (heavy alcohol use with chronic liver disease at cirrhosis stage, recent (< 8 weeks) or ongoing alcohol consumption, no other cause of liver failure) and biological (elevated bilirubin (> 50 µmol/L) and transaminase levels (AST > 50 U/L) with compatible AST to ALT ratio (> 1.5)) criteria, according to current guidelines issued by the NIAAA-funded Alcoholic Hepatitis Consortia [3].

### Data collection

Data were collected retrospectively by local investigators using electronic case report forms, then centralized and anonymized. On top of usual patient characteristics and medical history, data collected included social environment (marital status, employment), declared alcohol consumption, and liver disease characteristics (known underlying cirrhosis (and if so since when) or inaugural ARH, Child-Pugh score at hospital admission, registration on transplant list).

Data recorded at ICU admission included patient organ failures, with patient severity evaluated using the Simplified Acute Physiology Score (SAPS II), the Sequential Organ Failure Assessment (SOFA) score, and the Maddrey and MELD scores (we targeted patients with Maddrey scores ≥ 32). Acute-on-Chronic Liver Failure (ACLF) at ICU admission was also recorded, as well as ACLF grade, although this parameter was estimated only during data collection as ACLF was described towards the end of the study period [11]. In cases where corticosteroids therapy was initiated, Lille score at day seven was collected. Disease management was also recorded, both specifically concerning alcohol related hepatitis (liver biopsy, corticosteroids therapy, registration on transplant list, liver transplant) but also regarding general ICU care including use of support therapy (antibiotics, non-invasive and invasive ventilation, renal replacement therapy, vasopressors). Concerning hydrocortisone as part of a septic shock treatment, as the dose received (200 mg daily, equivalent to 50 mg of prednisone) somewhat matched the treatment dose in case of ARH (40 mg of prednisone), we decided to include analyze these patients as being treated by corticosteroids in our analysis. Finally, we recorded complications during care including gastrointestinal bleeding, encephalopathy (defined as lethargy or impaired consciousness with no other triggering factor identified other than liver failure (no blood ammonia dosage required)), ascites, hepatorenal syndrome (defined as acute renal failure with oliguria in absence of another kidney aggression factor, with urine sodium < 20 mmol/L and proteinuria < 0.5 g/day, and lack of response after fluid expansion), thrombosis and infection (proven by microbiological sampling or suspected due to fever or hypothermia and/or elevation of inflammation markers and/or onset or aggravation of hemodynamic instability with no other clinical explanation). All authors had access to the study data and reviewed and approved the final manuscript. Quality was assessed according to the STROBE checklist.

### Statistical analysis

Continuous variables are described as median and interquartile range (IQR) or mean (± SD) and compared using Wilcoxon’s rank sum test; categorical variables are summarized as counts (percent) and compared using Fisher’s exact test.

The primary outcome was in-ICU mortality. We performed a competing risk survival analysis with liver transplantation as a competing event. We used Fine and Gray’s model to assess factors associated with mortality. Variable selection was conditional based upon *P* value (entry value *p* < 0.2, exit value *p* > 0.1). Correlation and interaction were carefully checked as well as the log-linearity of continuous variables and proportional hazard assumptions. Results are given as sub-distribution hazard ratios (SHR; 95%CI).

To investigate the effect of corticosteroids used on ICU survival, only patients who received corticosteroids therapy no longer than three days prior to ICU admission were considered in the analysis. This limit was chosen as to specifically assess the effect of corticosteroids therapy regarding ICU patients’ trajectories, as authors felt that corticosteroids received prior than three days before ICU admission may have impacted patient morbidity and mortality regardless of ICU management. Cause-specific Cox models were used to estimate the effect of corticosteroid use on ICU survival, with unadjusted and adjusted (on age, sepsis at ICU admission, bilirubin level, modified SOFA score without bilirubin item, albumin level, and INR) comparisons. Then, to take into account allocation bias, we performed a propensity score overlap weighting analysis. Briefly, this strategy allows weighting patients from each treatment group with the probability to be assigned to the other treatment group. This allows assigning a higher weight to patients with intermediate-risk, and a lower weight to outliers, in both treatment groups. The analysis emphasizes the proportion of the population where the most treatment equipoise exists in clinical practice. This model has demonstrated high stability and the ability to obtain precise adjustment in various situations. The propensity score was built using logistic regression including variables associated with both corticosteroids use and outcome (i.e. age, coma, shock, acute kidney failure, acute respiratory failure, sepsis, SOFA score without total bilirubin, MELD score, albumin level, arterial lactate, total bilirubin, and prothrombin time). The quality of matching was assessed using standardized mean differences before and after weighting. The effect of corticosteroids use on mortality was then assessed using cause-specific Cox model with robust variance estimator and cumulative incidence functions were plotted. Because missing data accounted for less than 10% of our cohort, no imputation methods were used.

All tests were two-sided and *p*-values lower than 5% were considered to indicate significant associations. Analyses were performed using R statistical platform, version 3.0.2 (https://cran.r-project.org/*).*

## Results

### General characteristics

One hundred and eighty-seven patients were included in 9 French ICUs (6 tertiary hospitals, 2 general hospitals and 1 private hospital) over a 12-year period (Additional files 1 and 2, supplementary information). Table 1 reports the main characteristics at baseline. The median age was 53 [43–60] with 69% of men. 74% of patients were unemployed, and 55% were single. A prior diagnosis of cirrhosis was known in 40% of cases, and patients had a median declared daily alcohol intake of 112 g [60–200]. The median Child-Pugh score at hospital admission was 12 [11–13]. The main reasons for ICU admission were impaired consciousness (71%), sepsis (64%) (with 24% of total patients presenting spontaneous bacterial peritonitis), shock (44%), respiratory failure (37%), or hemorrhage (8%). A liver biopsy confirmed alcohol related hepatitis in 51% of cases.

**Table 1:** General characteristics of the 187 patients included. MELD, model for End-stage liver disease; ACLF, Acute-on-Chronic liver failure

Parameters	*N* (%) or Med. (IQR)
Age	53 [43;60]
Male gender	129 (69)
BMI	25.3 [23.2;28.8]
Marital status (single)	102 (55)
Unemployed	133 (74)
Type of admission:	
- in hospital - paramedics / emergency ward	123 (66) 64 (34)
Delay from hospital to ICU admission (days)	2 [0;7]
***Comorbidities***	
Smoking	100 (56)
Diabetes mellitus	19 (10)
Metabolic syndrome	44 (24)
Chronic kidney disease	3 (2)
***Liver disease status***	
Declared daily alcohol intake (g)	112 [60;200]
Cirrhosis known before ARH	75 (40)
Cirrhosis duration before ARH when known (years)	2 [1;4]
Child-Pugh score at hospital admission MELD score at ICU admission ACLF at ICU admission: - grade 1 - grade 2 - grade 3	12 [11;13] 31 [26;40] 154 (82) 18 (9) 56 (30) 80 (43)
Liver biopsy confirming ARH	95 (51)
***Treatment at hospital admission***	
Diuretics	35 (19)
Beta blockers	44 (24)
Aspirin	8 (4)
Anticoagulants	3 (2)
Proton pump inhibitors	43 (23)
***Reason for ICU admission (multiple possible)***	
Impaired consciousness	133 (71)
Sepsis	119 (64)
Shock	82 (44)
Respiratory failure	70 (37)
Hemorrhage	15 (8)

### Organ failure severity at ICU admission

Patient severity at admission was high, with a median SOFA score of 10 [7–13] and SAPS II of 52 [39–74]. At ICU admission, serum creatinine was 128 µmol/L [76–229], total bilirubin level 270 µmol/L [145–444], and arterial lactate 2.8 mmol/L [1.8–5.1]. 82% of patients presented ACLF at ICU admission, mostly grade 3 (43% of patients) and grade 2 (30% of patients). Within the first 24 h following ICU admission, 30% of patients required vasopressors, 16.5% invasive mechanical ventilation, and 18.5% renal replacement therapy (Table 2).

**Table 2:** Patient severity at admission. *SOFA*,* sequential organ failure assessment; SAPS II*,* simplified acute physiology score; INR*,* international normalized ratio; ACLF*,* acute-on-Chronic liver failure*

Parameters	*N* (%) or Med. (IQR)
Glasgow Coma Scale score	14 [6;15]
Mean arterial pressure (mmHg)	78 [70;92]
Heart rate (/min)	96 [82;112]
Respiratory rate (/min)	22 [17;28]
SOFA score	10 [7;13]
SAPS II	52 [39;74]
Leucocytes (Giga/L)	13.3 [8.4;21.3]
Platelets (Giga/L)	88 [59;127]
Hemoglobin (g/dL)	9.2 [8;10.9]
Serum sodium (mmol/L)	135 [131;139]
Serum creatinine (µmol/L)	128 [76;229]
Arterial pH	7.4 [7.3;7.5]
PaO_2_ (mmHg)	90 [73;116]
PaCO_2_ (mmHg)	30 [26;35]
Arterial lactate (mmol/L)	2.8 [1.8;5.1]
Blood aspartate aminotransferase (U/L)	141 [85;206]
Blood alanine aminotransferase (U/L)	56 [32;90]
Blood alkaline phosphatase (U/L)	154 [118;202]
Blood gamma-glutamyl transpeptidase (U/L)	232 [127;414]
Total blood bilirubin (µmol/L)	270 [145;444]
INR	2.7 [2.1;3.7]
Invasive mechanical ventilation (first 24 h)	31 (16.5)
Use of vasopressors (first 24 h)	56 (30)
Need of extra-renal replacement therapy (first 24 h)	12 (6.5)

### Patient outcomes

During the ICU stay, the main liver-related complications were encephalopathy (76%), ascites (67%), and hepatorenal syndrome (60%). 32% of patients acquired an infection, most commonly ventilator-associated pneumonia in 31% of ventilated patients, and urinary tract infections in 11% of ICU patients. Through ICU stay, 63% of patients received invasive mechanical ventilation, 62% vasopressors, and 36% renal replacement therapy. Liver transplantation was performed in 16 patients (8.5%) during hospital stay (with a median delay of 10 [4–24] days from ICU admission to liver transplant), while 4 patients (2%) alive at hospital discharge underwent liver transplantation later on. In-ICU and hospital mortality rates were 37% and 53% respectively (Table 3). Altogether, mortality rate was 15% (3/20) for patients receiving liver transplantation, and 58% (97/167) in non-transplanted patients.

**Table 3:** ICU management and patient outcome. HRS-AKI: hepatorenal Syndrome– Acute kidney injury

Parameters	*N* (%) or Med. (IQR)
Gastrointestinal bleeding	70 (37)
Encephalopathy	142 (76)
Ascites	125 (67)
Hepatorenal syndrome (HRS-AKI)	112 (60)
Deep vein thrombosis (excluding portal vein)	6 (3)
Atherothrombotic event (stroke, cardiac, mesenteric)	8 (4)
Infection during ICU stay (all)	59 (32)
Ventilator-associated pneumonia	36 (31)
Central line-associated bloodstream infection	14 (7)
Urinary tract infection	20 (11)
Treatment with vasopressors	115 (62)
Vasopressors treatment duration (days)	3.2 [1.3;8.3]
Invasive mechanical ventilation	117 (63)
Invasive mechanical ventilation duration (days)	7 [3;13]
Renal replacement therapy	68 (36)
Liver transplant: - during ICU/hospital stay - after ICU/hospital stay	20 (11) 16 (8.5) 4 (2)
Delay from ICU admission to liver transplant	10.5 [4;24]
Alive at ICU discharge	118 (63)
Alive at hospital discharge	87 (47)
Length of ICU stay (days)	7 [3;15]
Length of hospital stay (days)	16 [7;32]

### Factors associated with ICU mortality

**Fig. 1:**
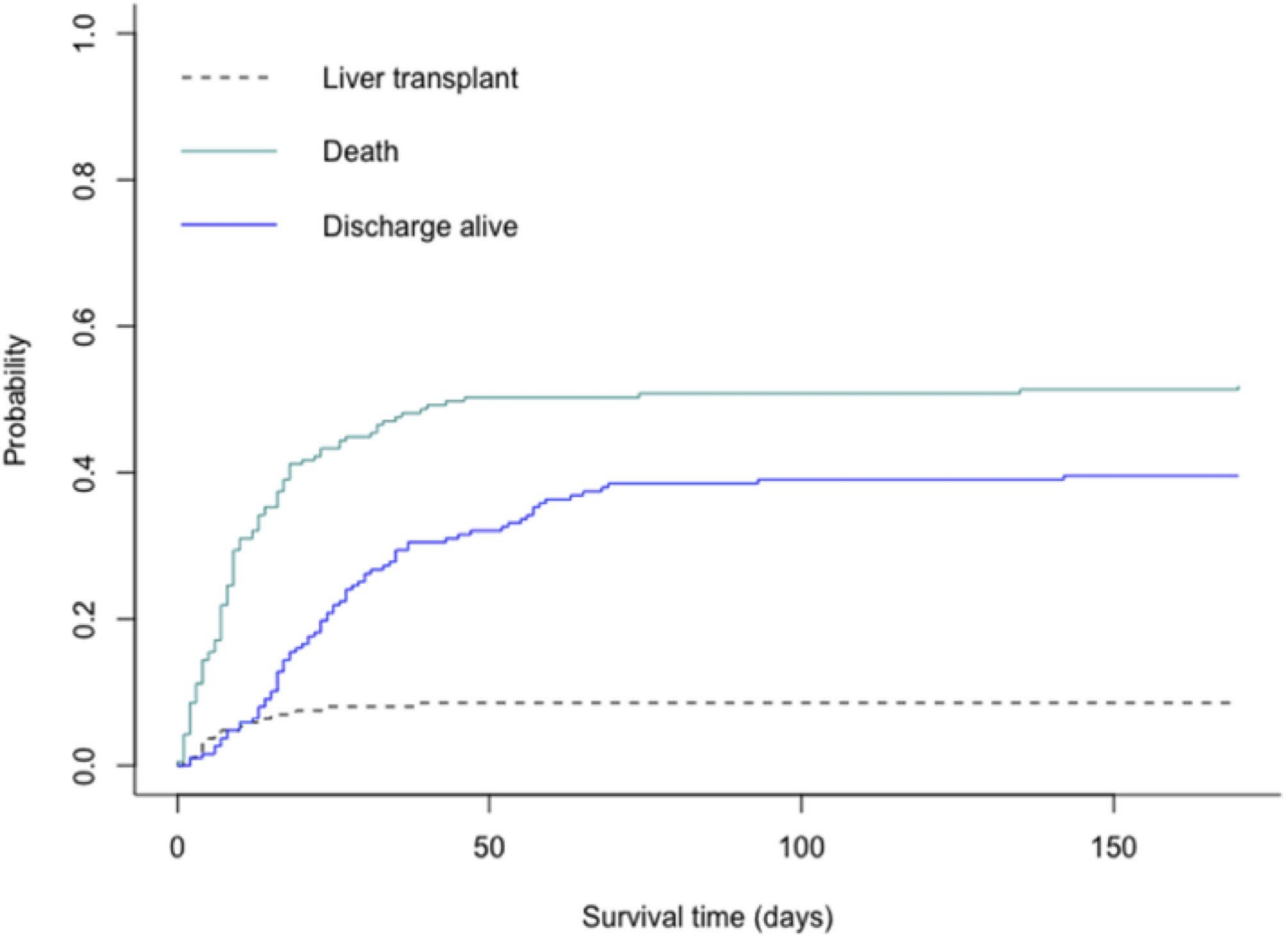
Population survival analysis (ICU death / liver transplant). ARH, Alcohol Related Hepatitis

**Fig. 2:**
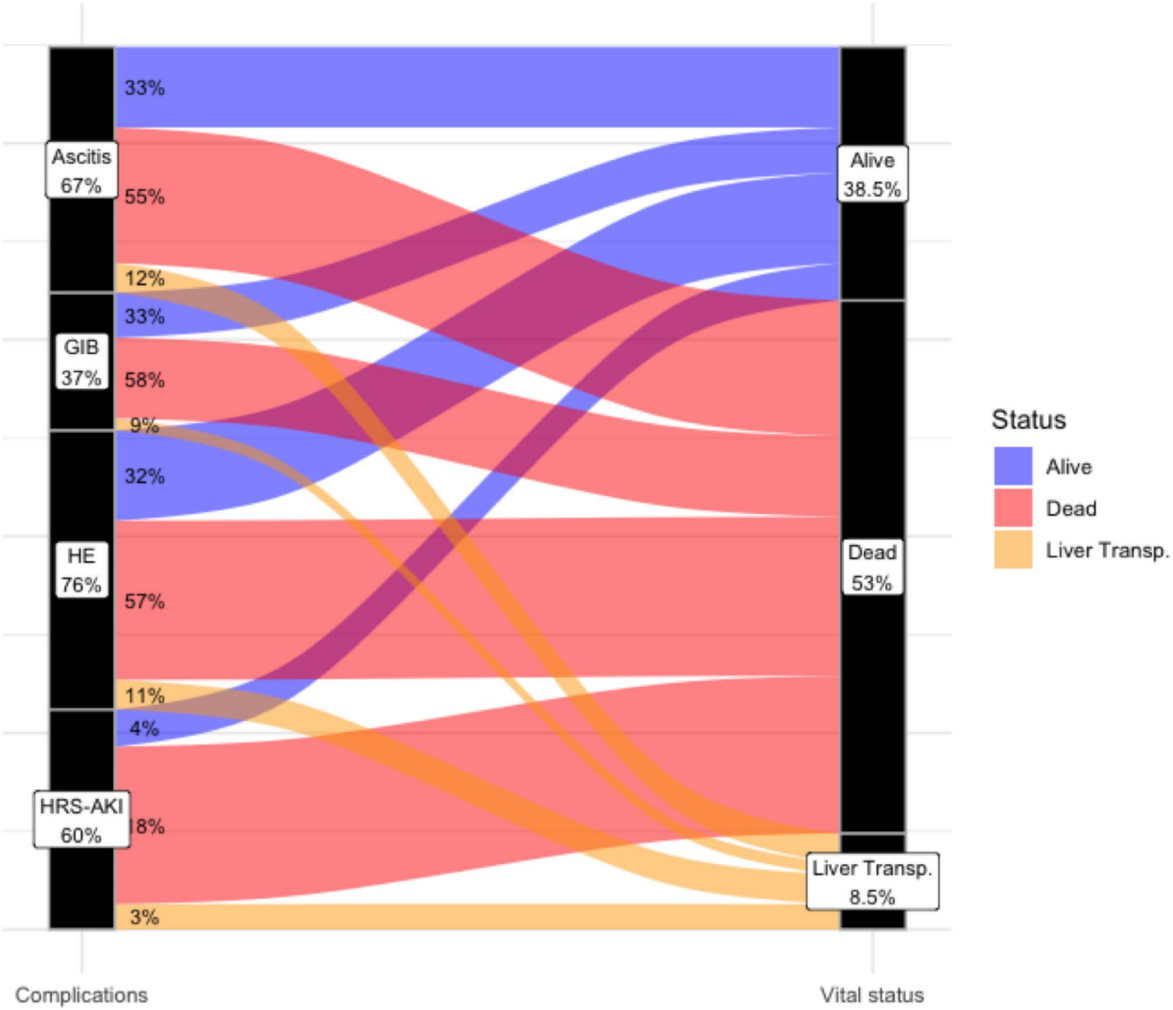
Status at ICU discharge by liver complications. GIB, Gastro-Intestinal Bleeding; HE, Hepatic Encephalopathy; HRS-AKI, Hepatorenal Syndrome– Acute Kidney Injury

Figure 1 depicted the cumulative incidence of death through ICU stay. Results from univariate analysis were presented in additional file 3 (supplementary information). By multivariable analysis, factors associated with mortality were modified SOFA score at admission (without total bilirubin) (SHR 1.08 [1.02–1.14] per one-point increase), arterial lactate (SHR 1.08 [1.03–1.13] per 1 mmol/l increase) and MELD score (SHR 1.09 [1.04–1.14] per 1 point increase), while employment was associated with increased survival (SHR 0.49 [0.28–0.86]). Patient trajectories according to main liver complications during follow-up were depicted in Fig. 2. As shown, hepatorenal syndrome (HRS-AKI) was the most common reason for death, carrying 71% of mortality.

### Alcohol related hepatitis management and effect of corticosteroids therapy

The median MELD score at ICU admission was 31 [26–40]. We compared patients who received corticosteroids (prednisolone or prednisone, 40 mg daily during 9 [7–28] days) with patients who did not, specifically patients for which corticosteroids therapy was initiated no longer than three days before ICU admission. Therefore, 82 patients receiving treatment were compared to 58 patients who did not (with 47 patients not included in the analysis as the delay between corticosteroids therapy initiation and ICU admission was ≥ 4 days). On the day of corticosteroids initiation, total bilirubin level was 279 µmol/L [162–455] and Maddrey score was 73 [56–84] (additional file 4). Patients with corticosteroids therapy had a less severe cirrhosis with lower Child-Pugh score (12 [11–13] vs. 13 [11–14], *p* = 0.043) and lower MELD score (28 [25–37] vs. 34 [28–40], *p* = 0.004). In addition, patients who have received corticosteroids had less severe organ failure at ICU admission, with lower arterial lactate levels (2.1 [1.8–3.1] vs. 3.1 mmol/L [2-7.2], *p* = 0.001) and lower SOFA score (8 [6–11] vs. 12 [9–15], *p* < 0.001). Sepsis was less frequent in corticosteroids-treated patients (49% vs. 72%, *p* = 0.006). Interestingly, a liver biopsy was more frequently done in patients receiving corticosteroids (68 vs. 22%, *p* < 0.001).

By univariate analysis, we did not find any significant effect of corticosteroids therapy on ICU survival (cause-specific hazard ratio: 0.73 [0.41–1.29], *p* = 0.279). This result did not change after adjustment on patients’ characteristics and severity (adjusted cause-specific hazard ratio: 0.96 [0.48–1.91], *p* = 0.904). Similarly, after propensity score weighting (additional files 5 and 6, supplementary information), corticosteroids therapy did not affect ICU survival (Cause-specific Hazard ratio: 1.13 [0.58–2.22], *p* = 0.714). Additional file 7 shows the cumulative incidence of death according to corticosteroids therapy on the weighted sample. Median delay between ICU admission and corticosteroids therapy initiation was 3 days, and no difference was found when comparing early (≤ 3 days) vs. delayed (> 3 days) treatment initiation (adjusted cause-specific HR [95%CI] = 0.73 [0.38–1.42] vs. 0.94 [0.50–1.76] respectively).

**Fig. 3:**
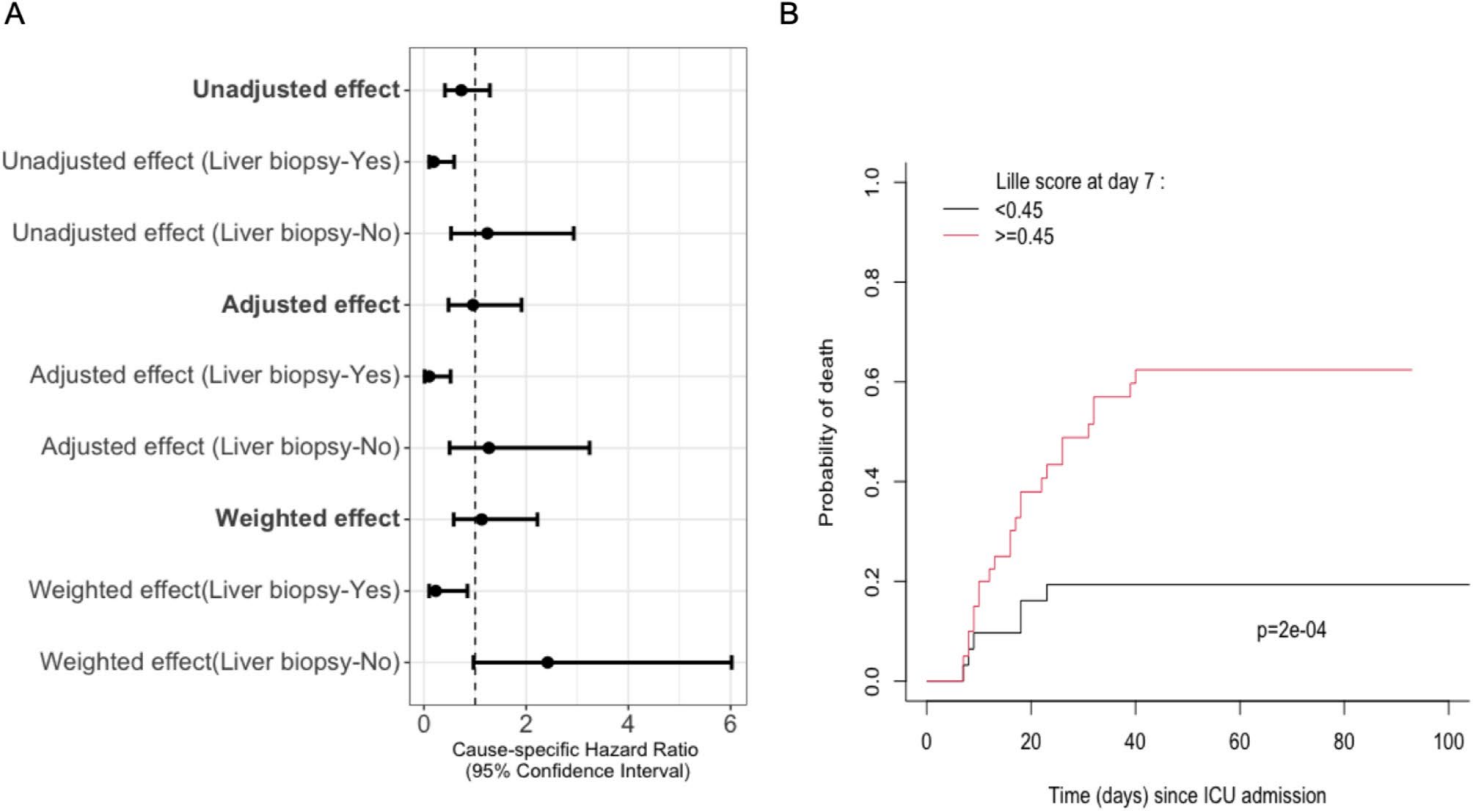
**3a.** Effect of corticosteroids therapy in biopsied and non-biopsied patients. **3b.** Patient outcome according to Lille score at day 7

We then analyzed the effect of corticosteroids therapy after separating those same patients in two groups, depending on whether a liver biopsy had been performed or not. An overall significant beneficial effect of corticosteroids therapy on ICU survival was found in patients in which a liver biopsy had been performed, and this effect remained in the adjusted and weighted comparisons (Fig. 3a). Patients in which a liver biopsy was not performed were more severe (higher Maddrey, MELD and SOFA scores, additional file 8). Among patients treated with corticosteroids (82 patients), we analyzed survival according to day-7 Lille score [7]. We found that corticosteroids responders, defined as Lille score < 0.45 at day 7, had significantly better survival when compared to non-responders (Fig. 3b).

Finally, when separating our population in two subgroups (< 2011 and ≥ 2011, year when liver transplantation was confirmed as a possible treatment option for non-responders to corticosteroids therapy [9]), our study did not find any significant effect between year of ICU admission and mortality.

## Discussion

We here describe the characteristics of 187 patients with alcohol related hepatitis admitted in 9 ICUs in France over a 12-year period. We found that ARH patients had severe organ failure with frequent use of organ support therapy and ultimately high mortality. We identified several independent factors predictive of ICU mortality at ICU admission, including SOFA score, arterial lactate level and MELD score, while employment was a protective factor. Corticosteroids therapy was associated with a better outcome in responders according to Lille score.

We performed an original large-scale multicentric study concerning ARH patients specifically in ICUs, both in tertiary and general hospitals across several geographic regions, in order to make these results generalizable. ARH diagnostic was still largely made combining clinical and biological criteria, sometimes combined with histological proof. Here, liver biopsy was performed in only half of cases. Liver biopsy is not mandatory for ARH diagnosis but in critically ill patients, histological confirmation should be obtained because of several potential confounders (i.e., shock, gastrointestinal bleeding, sepsis, drugs impacting liver function or blood test results [12]). US guidelines on ARH therefore clearly state that a liver biopsy is required when facing these potential confounding factors (as when treating ICU patients), but this is not applied widely enough, as shown in our study. Interestingly, in most cases (60%), the underlying cirrhosis was diagnosed at the time of the ARH episode.

In our study, fatal outcome of critically ill ARH patients was very frequent with a mortality rate of 37% in ICU and 53% in hospital, higher than in previous studies on severe ARH not focused on ICU patients [2], which underlines the upmost severity of these specific ARH patients. Our outcome results are in line with other studies reporting global mortality of ICU cirrhotic patients around 40%, although their prognosis has significantly improved in the last decades [13, 14]. The high rate of grade 2 and 3 ACLF in these patients may account for such high mortality. As in our study, acute kidney injury and specifically hepatorenal syndrome is one of the most severe complications of end-stage cirrhosis, with recent studies finding mortality rates without liver transplantation of up to 80% [15, 16].

Our study found no overall benefit to corticosteroids therapy concerning ICU and in-hospital mortality. However, it is now well established that corticosteroids’ benefit varies from one patient to another, “responders” identified using Lille score at day 7 [7, 17]. This remains highly relevant when dealing with ICU patients in our study, with mortality here differing significantly between responders and non-responders as defined by a standard Lille score cutoff of 0.45 [7]. Therefore, ICU physicians should use this tool to early distinguish patients benefiting from corticosteroids therapy from those where it should be stopped to avoid adverse events, mainly infectious complications. Although no effect was found between early and delayed treatment in our study, we cannot rule out that delays in corticosteroids therapy may have resulted in a missed therapeutic window, therefore affecting our results concerning their overall effect on mortality.

In our study, corticosteroids therapy was not initiated in nearly one third of patients with a theoretical indication, with several possible explanations. First, ARH may not have been considered a possible initial diagnosis in all patients. Second, even in cases when ARH was considered a possible diagnosis, access difficulties to liver biopsy may have refrained physicians from using corticosteroids without histological proof. Finally, corticosteroids may have not been used either because patients were deemed too severe, or because of uncontrolled sepsis. This hypothesis could partly explain why a beneficial effect of corticosteroids therapy was found in the subgroup of patients that underwent liver biopsy (those patients being less severe), as physicians might not have performed a biopsy in the most severe patients considering they would not initiate corticosteroids therapy even in case of histologically proven ARH. Overall, our study underlines the importance of performing a liver biopsy specifically in the setting of ICU patients, as recommended in current guidelines [3].

Early liver transplantation has emerged in the last decade as a possible treatment for carefully selected patients not responding to corticosteroids therapy [9]. It remains possible even when patients require ICU care, with 16 patients undergoing liver transplantation in our study. With significantly increased survival rates and alcohol relapse rates comparable to those of patients undergoing liver transplant for alcohol related cirrhosis outside of ARH [10], this treatment option should always be considered, and discussion with tertiary centers performing liver transplant is imperative in cases of lack of response to corticosteroids. Recent multicentric studies, both in Europe and the United States, concur in finding excellent outcomes in patients with severe ARH and/or ACLF-3, with survival rates similar to that of patients with lower ACLF grades or no ACLF, which stresses the importance of patient referral to tertiary centers for evaluation and possible liver transplant [10, 18, 19]. These studies also underline a short “transplantation window”, indicating that patients should be referred early to tertiary centers, as complications appearing later-on during patient care could jeopardize not only liver transplantation feasibility, but also patient’s ability to consent to such a treatment due to impaired consciousness. Our study did not find an effect of year of ICU admission (< or ≥ 2011) and ICU mortality, although it was not designed to answer this specific question.

Our study has several limitations. First, because of its observational retrospective nature, only association and not causality may be inferred. Second, only few of our patients (< 10%) solely presented liver dysfunction at ICU admission, with most patients presenting both severe ARH and other causes for ICU admission (e.g. sepsis, neurological impairment, hemorrhage). Therefore, we were not able to separate patients admitted for ARH-related complications, or for causes unrelated to ARH. Third, because early liver transplantation appeared as a possible treatment option in the middle of our study period (2011), its impact on patient management and mortality could not be precisely depicted here. This could also account for the relatively low number of patients who underwent liver transplantation. Similarly, ACLF could not be studied in detail in our study, as the concept of ACLF was described a few years after the beginning of our study period. Overall, the changes in ARH patients management in the last decades (from early change in bilirubin levels [20] and Lille score, to the description of liver transplantation in those patients, and now the generalization of this treatment option) combined with our relatively large study period (2006 to 2017) accounts for the heterogeneity of certain practices and treatment options. Fourth, it has been proposed that Lille score calculated on day 4 of corticosteroids treatment has comparable accuracy to the one calculated on day 7 to predict response to corticosteroids as well as 28- and 90-day mortality [21]. Unfortunately, we lacked the data to assess day 4 Lille score accuracy in our population. Finally, our study lacks data collection concerning the reasons why patients not responding to corticosteroids therapy were not considered for an early liver transplantation.

## Conclusion

In this large multicenter ICU study, we found that alcohol related hepatitis is a highly severe condition leading to high mortality in critically ill patients. Mortality was mainly driven by organ failure at admission as well as hepatorenal syndrome occurrence during ICU stay. Liver biopsy should be performed in such patients to confirm alcohol related hepatitis diagnosis, as many confounding factors exist in ICU patients. Corticosteroids use might be associated with improved outcome in a subset of patients. In non-responders, liver transplantation is a possible therapeutic option in selected patients, with excellent results. Patients should therefore early be referred to tertiary centers to discuss liver transplantation.

## Electronic supplementary material

Below is the link to the electronic supplementary material.

**Supplementary Material 1**: **Additional file 1**: Study flow chart. ARH, Alcohol Related Hepatitis. **Additional file 2**: Number of alcohol related hepatitis patients included in each center. **Additional file 3**: Predictive factors of ICU mortality (univariate analysis). SOFA, Sequential Organ Failure Assessment; SAPS II, Simplified Acute Physiology Score; MELD, Model for End-stage Liver Disease; INR, International Normalized Ratio. **Additional file 4**: Comparison between patients treated and not treated with corticosteroids (only patients for which corticosteroids were initiated no more than three days prior to ICU admission were included). SOFA, Sequential Organ Failure Assessment; SAPS II, Simplified Acute Physiology Score; INR, International Normalized Ratio; ARH, Alcohol Related Hepatitis. **Additional file 5**: Propensity score distribution, before and after weighting. **Additional file 6**: Balance checks after weighting on propensity score for corticosteroids use: absolute standardized mean difference before and after weighing (panel A) and cumulative frequencies of main continuous predictors (panel B). PT, Prothrombin Time; SOFA, Sequential Organ Failure Assessment; MELD, Model for End-stage Liver Disease; ARF, Acute Respiratory Failure; AKI, Acute Kidney Failure. **Additional file 7**: Cumulative incidence of death according to corticosteroids therapy on the weighted sample. **Additional file 8**: Comparison between patients in which a liver biopsy was or was not performed (only patients for which corticosteroids were initiated no more than three days prior to ICU admission were included). ARH, Alcohol Related Hepatitis; INR, International Normalized Ratio; SOFA, Sequential Organ Failure Assessment; SAPS II, Simplified Acute Physiology Score; MELD, Model for End-stage Liver Disease.

## Data Availability

The datasets used and/or analyzed during the current study are available from the corresponding author upon reasonable request.

## Abbreviations

ARH: Alcohol Related Hepatitis
ICU: Intensive Care Unit
SOFA: Sequential Organ Failure Assessment
MELD: Model for End-stage Liver Disease
AST: Aspartate Aminotransferase
ALT: Alanine Aminotransferase
INR: International Normalized Ratio
SAPS II: Simplified Acute Physiology Score
ACLF: Acute-on-Chronic Liver Failure
HRS-AKI: Hepatorenal Syndrome– Acute Kidney Injury
