# Supplementary material for: Alcohol related hepatitis in intensive care units: clinical and biological spectrum and mortality risk factors: a multicenter retrospective study
Source: Ann Intensive Care. 2025 Apr 15;15:53. doi: 10.1186/s13613-025-01450-2 (PMC11996726; doi:10.1186/s13613-025-01450-2)
Supplement: Supplementary file 1 — Supplementary Material 1: Additional file 1: Study flow chart. ARH, Alcohol Related Hepatitis. Additional file 2: Number of alcohol related hepatitis patients included in each center. Additional file 3: Predictive factors of ICU mortality (univariate analysis). SOFA, Sequential Organ Failure Assessment; SAPS II, Simplified Acute Physiology Score; MELD, Model for End-stage Liver Disease; INR, International Normalized Ratio. Additional file 4: Comparison between patients treated and not treated with corticosteroids (only patients for which corticosteroids were initiated no more than three days prior to ICU admission were included). SOFA, Sequential Organ Failure Assessment; SAPS II, Simplified Acute Physiology Score; INR, International Normalized Ratio; ARH, Alcohol Related Hepatitis. Additional file 5: Propensity score distribution, before and after weighting. Additional file 6: Balance checks after weighting on propensity score for corticosteroids use: absolute standardized mean difference before and after weighing (panel A) and cumulative frequencies of main continuous predictors (panel B). PT, Prothrombin Time; SOFA, Sequential Organ Failure Assessment; MELD, Model for End-stage Liver Disease; ARF, Acute Respiratory Failure; AKI, Acute Kidney Failure. Additional file 7: Cumulative incidence of death according to corticosteroids therapy on the weighted sample. Additional file 8: Comparison between patients in which a liver biopsy was or was not performed (only patients for which corticosteroids were initiated no more than three days prior to ICU admission were included). ARH, Alcohol Related Hepatitis; INR, International Normalized Ratio; SOFA, Sequential Organ Failure Assessment; SAPS II, Simplified Acute Physiology Score; MELD, Model for End-stage Liver Disease. [file 13613_2025_1450_MOESM1_ESM.docx]

**
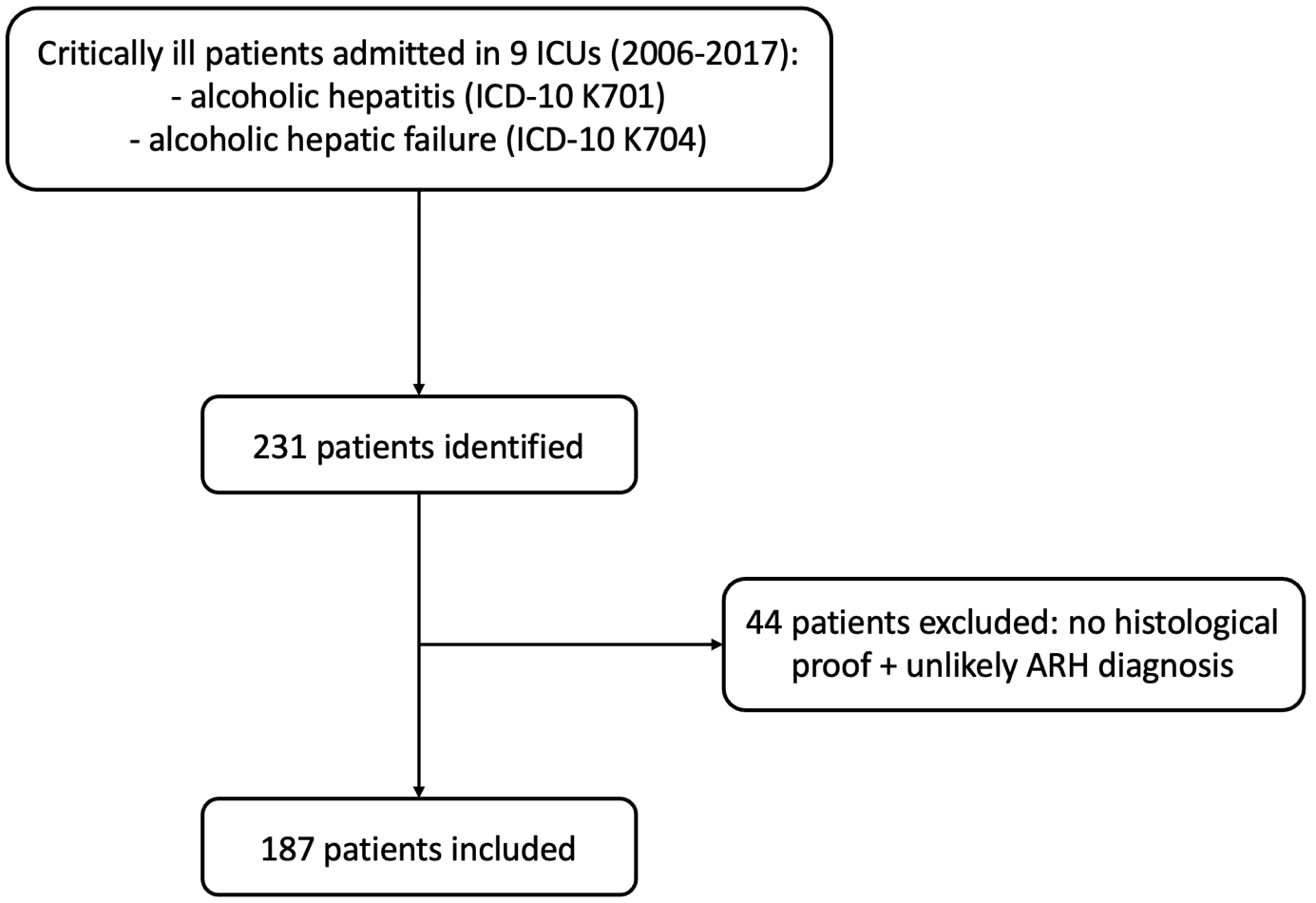
**

**Additional file 1. Study flow chart.** ARH, Alcohol Related Hepatitis

**Additional file 2.** Number of alcohol related hepatitis patients included in each center.

**Additional file 3. Predictive factors of ICU mortality (univariate analysis)**

**Additional file 4. Comparison between patients treated and not treated with corticosteroids** (only patients for which corticosteroids were initiated no more than three days prior to ICU admission were included). SOFA, Sequential Organ Failure Assessment; SAPS II, Simplified Acute Physiology Score; INR, International Normalized Ratio; ARH, Alcohol Related Hepatitis

**
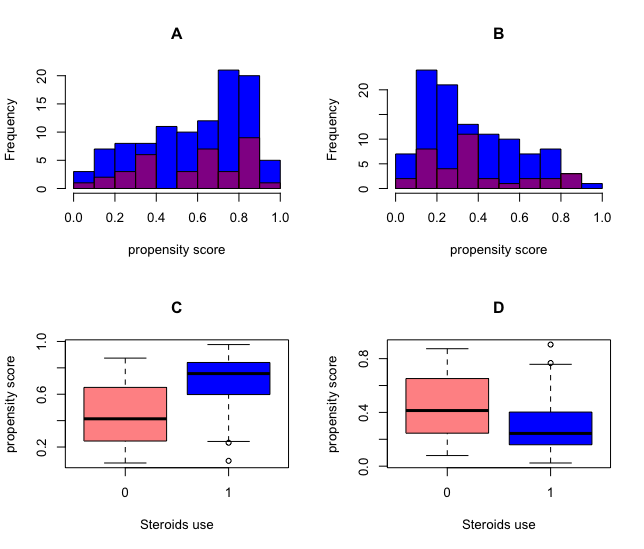
**

**Additional file 5. Propensity score distribution, before and after weighting.** Covariables used to create propensity score: patient age and ICU admission coma, shock, acute kidney failure, acute respiratory failure, sepsis, SOFA score without total bilirubin, MELD score, albumin level, arterial lactate, total bilirubin, and prothrombin time.


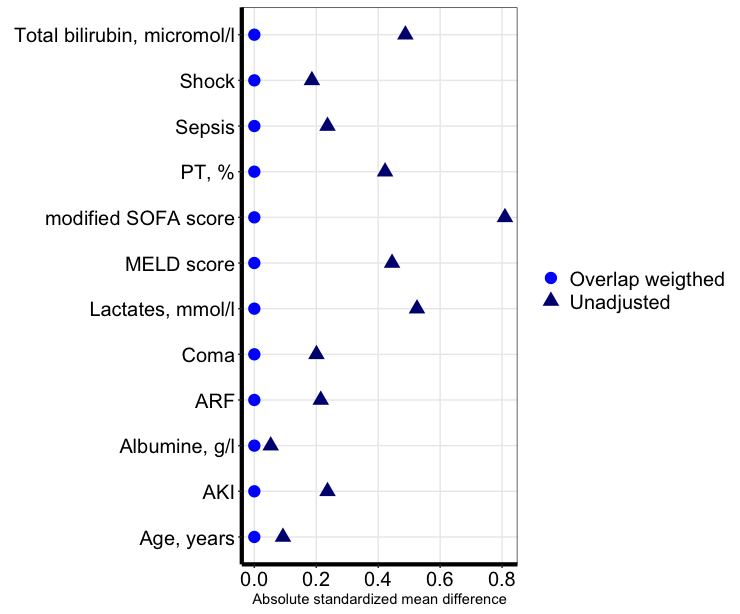

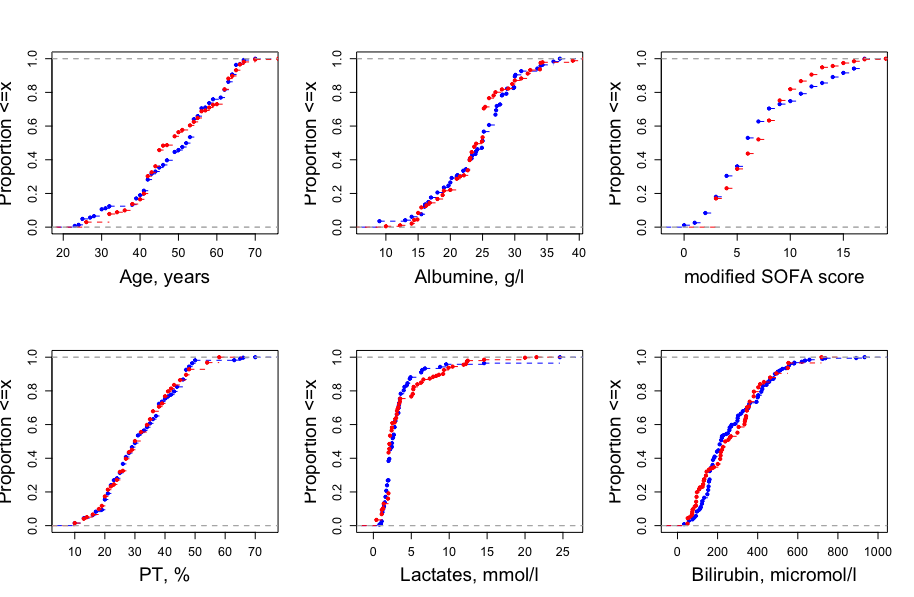


**Additional file 6. Balance checks after weighting on propensity score for corticosteroids use:** absolute standardized mean difference before and after weighing (panel A) and cumulative frequencies of main continuous predictors (panel B). PT, Prothrombin Time; SOFA, Sequential Organ Failure Assessment; MELD, Model for End-stage Liver Disease; ARF, Acute Respiratory Failure; AKI, Acute Kidney Failure

**
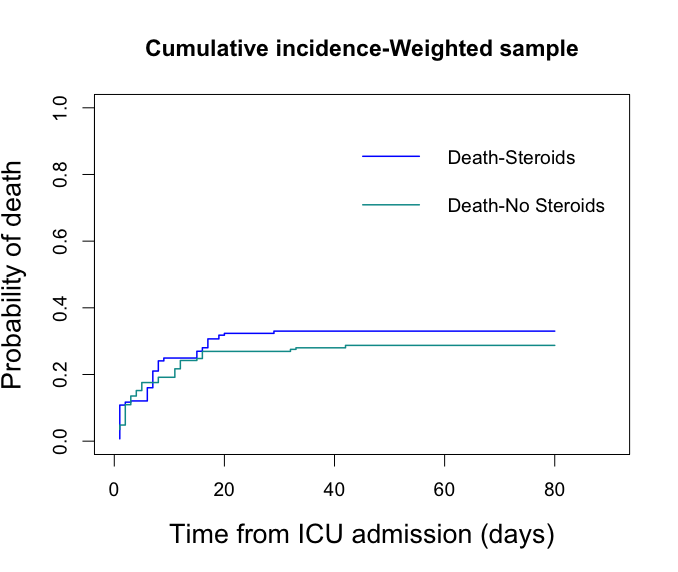
**

**Additional file 7. Cumulative incidence of death** according to corticosteroids therapy on the weighted sample.

**Additional file 8. Comparison between patients in which a liver biopsy was or was not performed** (only patients for which corticosteroids were initiated no more than three days prior to ICU admission were included). ARH, Alcohol Related Hepatitis; INR, International Normalized Ratio; SOFA, Sequential Organ Failure Assessment; SAPS II, Simplified Acute Physiology Score; MELD, Model for End-stage Liver Disease
